# Can Milk Teeth be Impacted? Why Not: A Case of Six Impacted Primary Teeth

**DOI:** 10.5005/jp-journals-10005-1270

**Published:** 2015-02-09

**Authors:** Josna Vinutha Yadiki, Yellamma Bai Kategari, Pujita Chada, Venu Vallakatla

**Affiliations:** Assistant Professor, Department of Pedodontics and Preventive Dentistry, Army College of Dental Sciences, Secunderabad, Andhra Pradesh India; Professor and Head, Department of Pedodontics and Preventive Dentistry, Army College of Dental Sciences, Secunderabad, Andhra Pradesh India; Assistant Professor, Department of Pedodontics and Preventive Dentistry, Army College of Dental Sciences, Secunderabad, Andhra Pradesh India; Assistant Professor, Department of Pedodontics and Preventive Dentistry, Army College of Dental Sciences, Secunderabad, Andhra Pradesh India

**Keywords:** Impacted primary teeth, Ectodermal, Surgical exposure.

## Abstract

The prevalence rate of impacted primary teeth is rare, still we can see impacted teeth in ectodermal dysplasia anhydrotic (EDA), endocrine deficiencies, metabolic disorders and local factors like cysts, tumors, trauma and thickened overlying bone or soft tissue. In cases of EDA, delayed tooth eruption is one of the characteristic finding.

Present case report related to a rare case of primary teeth impaction of a 3 years old male child along with EDA. Intraoral examination and radiographs confirmed impacted primary maxillary and mandibular centrals and mandibular lateral incisors. Treatment carried out was surgical exposure of impacted primary teeth, then after patient was followed up for regular visits to check eruption status of the teeth.

**How to cite this article:** Yadiki JV, Kategari YB, Chada P, Vallakatla V. Can Milk Teeth be Impacted? Why Not: A Case of Six Impacted Primary Teeth. Int J Clin Pediatr Dent 2014; 7(3):220-222.

## INTRODUCTION

The prevalence of ectodermal dysplasia anhydrotic (EDA) is unknown; however, the incidence in male is estimated at 1 in 100,000 births although the condition is usually overlooked in infants (Bergendal et al, 1998). Ectodermal dysplasia anhydrotic is characterized by the triad of signs comprising sparse hair, abnormal or missing teeth and inability to sweat due to lack of sweat glands.

The impaction can be caused by systemic or local etiologic factors. Other factors include: overlying cysts or tumors, trauma, thickened overlying bone or soft tissue.^1^

Present case with impacted maxillary and mandi-bular central incisors and mandibular lateral incisors along with EDA and thickened overlying soft tissues over the teeth is reported.

## CASE REPORT

**Fig. 1:**
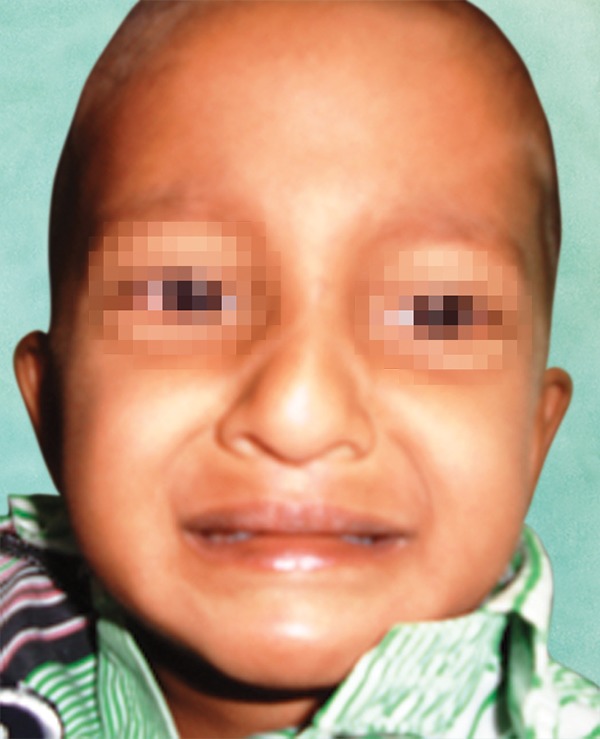
Clinical photograph of ectodermal dysplasia patient

A 3-year-old male child along with mother reported to the Department of pedodontics and preventive dentistry with a chief complaint of unerupted upper and lower anterior teeth (Fig. 1).

On general case history, it has been noted that the parents were consanguineously married couple with two children. The first child is 3 years old male born by full term normal delivery reported to the department. The second female child is of 6 months old. There was no history of birth injury.

On medical examination, patient had clinical signs of EDA like dryness of the skin, hypoplastic midface, sparse, fine, slowly growing scalp hair, sparse eye brows and eye-lashes. No other systemic disease was noted. There were no similar significant findings in his parents or sibling.

**Fig. 2:**
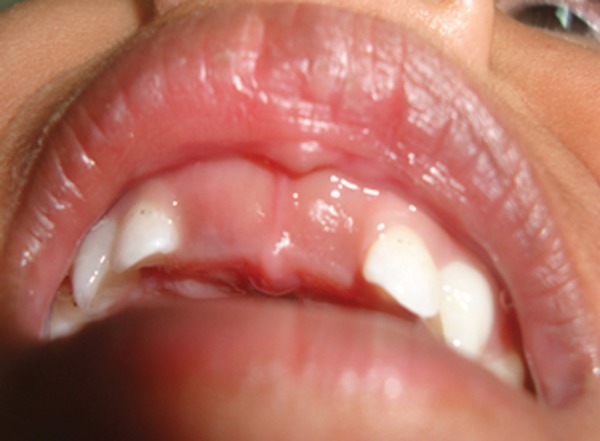
Intraoral photograph showing unerupted maxillary central incisors

Intraoral examination of the patient revealed unerup-ted 51, 61, 72, 71, 81 and 82 with overlying thickened fibrous tissue (Fig. 2). Seventy-five and 85 were erupting and other primary teeth were erupted normally.

**Fig. 3:**
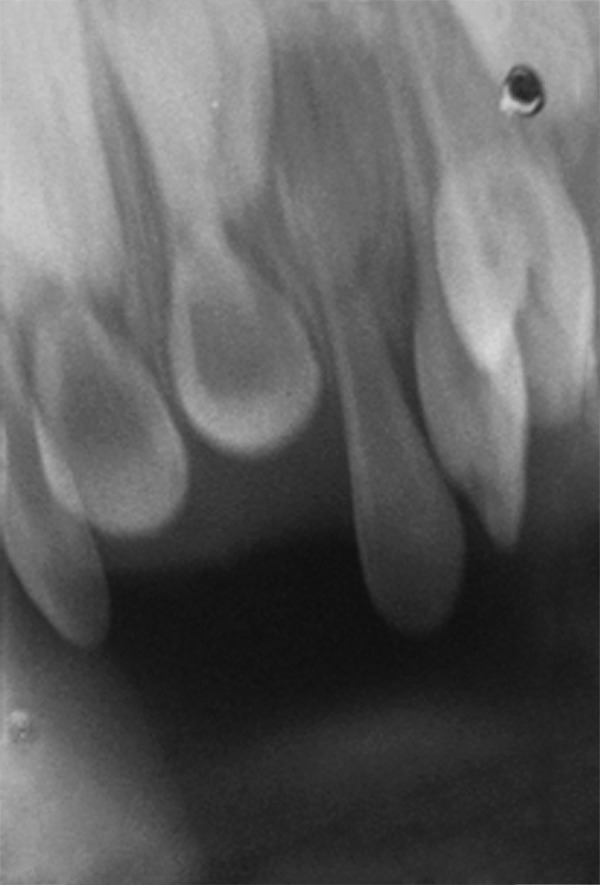
IOPA showing impacted primary maxillary central incisors

**Fig. 4:**
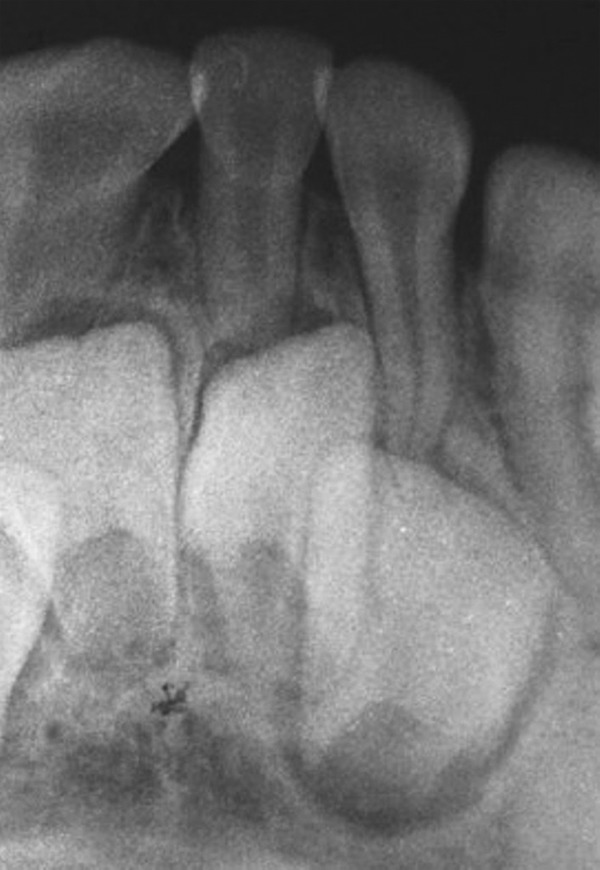
IOPA showing impacted primary mandibular central and lateral incisors

Intraoral periapical radiographs showed morphologically normal impacted primary maxillary central incisors (Fig. 3) and mandibular central and lateral incisors (Fig. 4). Permanent tooth buds were seen.

The case was diagnosed as X-linked hypohydrotic EDA with impacted primary anterior teeth covered by thickened fibrous tissue after evaluating all findings. Pedodontist should diagnose the condition as early as possible, so that surgical exposure can be performed in case of thickened soft tissue overlying the impacted teeth as performed in this case.

**Fig. 5:**
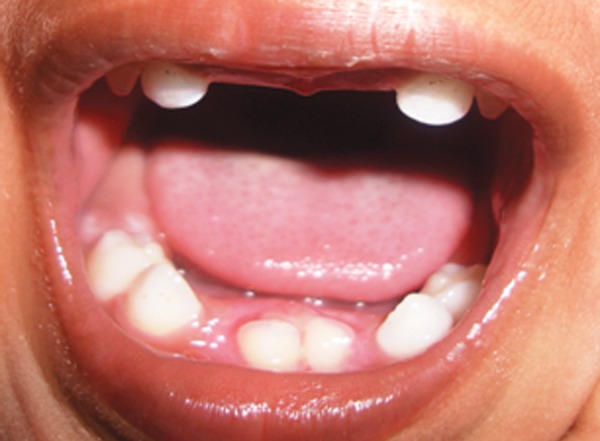
Postoperative mandibular central incisors

**Fig. 6:**
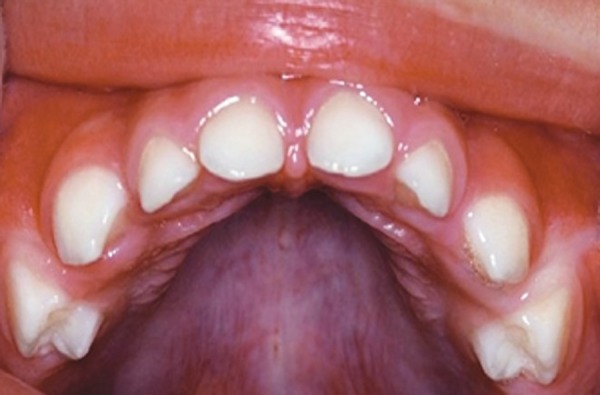
Postoperative maxillary central incisors

Informed consent was taken from the child's parents for treatment. Patient was on the lap of his parent. Patient was given topical followed by infltration local anesthesia (lidocaine with adrenaline 1:100,000). The procedure involved raising a mucoperiosteal fap, exposing the crowns of 71 and 81, but 72 and 82 were not exposed in the same visit, because they were 3 to 4 mm below the adjacent teeth on radiograph (Fig. 5). On second visit, the remaining 51 and 61 were surgically exposed (Fig. 6).

The child's recovery was normal and 51, 61, 71 and 81 were erupting normally. The patient was advised to come for regular checkup to monitor the eruption status of the teeth.

## DISCUSSION

Eruption is the continuous process of the tooth from its developmental location to its functional location. Tooth impaction refers to situations where failure to erupt because of physical barrier (impacted) or lack of eruptive force (embedded) appears to be due to a blocking and the tooth remains unerupted. The impaction can be caused by systemic or local etiologic factors.^2^

Systemic factors are endocrine deficiencies, organ developmental failures, metabolic disorders, drugs, nutritional deficiencies and some genetic factors. If teeth have not erupted in an infant during the first year, the underlying etiology may be related to one of the above mentioned factors.^3^

Even few local factors like thickened soft tissue and lack of eruption force might prevent primary tooth erup-tion.^4^

Other features of EDA are frontal bossing, prominent supraorbital ridge, saddle nose, ‘dished-in’ appearance of midface, broad and high cheek bones, pointed chin and everted lips.

In this case, patient had local cause of thickened soft tissue which has been evaluated clinically and radiographically. Patient had dryness of the skin and decreased sweating, depressed midface, sparse hair, sparse eyebrows and eyelashes and ‘old man’ facies appearance. These features concluded the diagnosis of hypohydrotic EDA.

The impaction of permanent mandibular third molars is the most common (82.5%), followed by maxillary third molars (15.6%) and maxillary canines (0.8%). The incidence of primary tooth impaction is twice as common in the mandible as in the maxilla. Primary molars are usually affected, impacted primary anterior teeth are rare.

An obstruction should be treated with an uncovering procedure that includes surgical exposure of the incisal edge as discussed in the case report.^5^

## CONCLUSION

Variation in the normal eruption pattern of sequence and timing of the teeth is a common finding. An impacted tooth is usually associated with permanent dentition but is rare during the development of primary dentition, with a reported prevalence ratio of 1:10,000.

Due to the systemic or local factors, if there is a delay or impacted eruption pattern for permanent or primary teeth, it is always advisable for early diagnosis and management in order to prevent later complications.

Impacted permanent or primary tooth is uncommon and if it is not treated it may cause ankylosis or ectopic eruption of permanent tooth leading toward developing malocclusion.

Treatment of impacted primary teeth is to enhance the esthetics, function and phonetics, normalize the vertical dimension and support the facial soft tissues.
